# Efficacy of Low-dose Ketamine for Control of Acute Pain in the Emergency Setting: A Systematic Review and Meta-analysis of Randomized Controlled Trials

**DOI:** 10.5811/westjem.2023.2.58368

**Published:** 2023-05-09

**Authors:** Mengyao Ying, Yuetun Zuo

**Affiliations:** Changxing People’s Hospital of China, Department of Emergency Medicine, Zhejiang Province, People’s Republic of China

## Abstract

**Introduction:**

Ketamine can be particularly helpful in situations where the clinician is not able to administer opioids and require an alternate analgesic, such as for patients who are already on high-dose opioids, have a history of addiction, or for opioid-naïve children and adults. In this review, our goal was to obtain a comprehensive estimate of the efficacy and safety of low-dose ketamine (dose less than 0.5 milligrams per kilogram or equivalent) compared to opiates for the control of acute pain in the emergency setting.

**Methods:**

We conducted systematic searches in PubMed Central, EMBASE, MEDLINE, the Cochrane Library, ScienceDirect, and Google Scholar from inception until November 2021. We used the Cochrane risk-of-bias tool to assess the quality of included studies.

**Results:**

We carried out a meta-analysis with a random-effects model and reported pooled standardized mean difference (SMD) and risk ratio (RR) with 95% confidence intervals depending on the type of the outcome. We analyzed a total of 15 studies with 1,613 participants. Half of them had high risk of bias and were conducted in the United States of America. The pooled SMD for pain score was −0.12 (95% CI −0.50–0.25; I2=68.8%) within 15 minutes, −0.45 (95% CI −0.84–−0.07; I2=83.3%) within 30 minutes, −0.05 (95% CI −0.41–0.31; I2=86.9%) within 45 minutes, −0.07 (95% CI −0.41–0.26; I2=82%) within 60 minutes, and after 60 minutes the pooled SMD was 0.17 (95% CI −0.07–0.42; I2=64.8%). The pooled RR for need of rescue analgesics was 1.35 (95% CI 0.73–2.50; I2=82.2%). The pooled RRs were as follows: 1.18 (95% CI 0.76–1.84; I2=28.3%) for gastrointestinal side effects; 1.41 (95% CI 0.96–2.06; I2=29.7%) for neurological side effects; 2.83 (95% CI 0.98–8.18; I2=47%) for psychological side effects; and 0.58 (95% CI 0.23–1.48; I2=36.1%) for cardiopulmonary side effects.

**Conclusion:**

Low-dose ketamine might have higher or equivalent efficacy and safety when compared to opioids for managing acute pain among patients presenting to the emergency setting. However, further studies are required to establish conclusive evidence, owing to the heterogeneity and poor quality of existing studies.

## INTRODUCTION

Acute pain is responsible for more than half of the visits to the emergency department (ED).^1^,^2^ Therefore, management of acute pain is an essential aspect of patient satisfaction and care. Currently, opioids are the most common group of analgesics used for the management of acute pain.^3^ However, given the complications associated with use of opioids, many patients would benefit from an opioid alternative for an effective and safer control of pain. In addition, certain categories of patients, such as opioid-naïve children and adults, the elderly, chronic users of opioid medications, patients with a history of opioid use disorder, and those using drugs for opioid use disorders or alcohol dependence, would also benefit from an effective alternative to opioids.^4^,^5^

Ketamine is a N-methyl-D-aspartate (NMDA) receptor antagonist drug with anaesthetic and analgesic properties.^6^ While traditionally it was used as an anaesthetic, it was replaced by the newer class of anaesthetics with better efficacy and minimal side effects. Over the past few years, ketamine has been used in the emergency setting for induction before intubation and procedural sedations, given its dissociative properties that allow preservation of the airway reflexes and hemodynamic stability properties.^7^

At the lower sub-dissociative doses (less than 0.5 milligrams per kilogram [mg/kg] intravenous [IV] doses), ketamine has been shown to have better analgesic property than opiates for the acute and chronic pain.[8]. Although, the use of ketamine for managing acute pain is a relatively novel concept, it has certain unique features that could prove advantageous in improving patient outcomes, particularly for the group of people mentioned above. Several studies have examined the role of ketamine compared to opioids for the management of acute pain.^9^–^11^ Although few reviews have attempted to summarized the findings of these reports, they have included a very limited number of studies and provided inconclusive evidence on the efficacy and safety of ketamine for acute pain management.^12^,^13^ Our main goal in this comprehensive systematic review and meta-analysis was to evaluate the role of low-dose ketamine compared to opiates for the management of acute pain in the emergency setting.

## MATERIALS AND METHODS

### Design

The protocol of the study was registered in PROSPERO, registration number CRD42021289270. In this systematic review, we used the “Preferred Reporting Items for Systematic Reviews and Meta-Analyses (PRISMA) statement 2020” for reporting meta-analyses.^14^

### Eligibility Criteria

#### Study Design

We included studies with any of the following study designs: parallel-arm individual or cluster randomized controlled trials (RCT). For cross-over trials, only the first half of the trial (before crossing over) were included. We included only published, full-text studies or abstracts, while excluding unpublished data or gray literature.

#### Participants

We included studies conducted in patients reporting to the emergency setting or ED with acute pain were included to form two groups (ketamine and control groups), irrespective of the cause of pain. We excluded studies conducted among postoperative patients.

#### Intervention and Comparison Groups

Studies using the IV low-dose ketamine (dose less than 0.5 mg/kg or equivalent) for the management of acute pain as intervention were included. The comparison group used opioids such as morphine, fentanyl, etc, or were placebo-controlled trials or standard care.

**Population Health Research Capsule**

What do we already know about this issue?

*At the lower sub-dissociative doses (< 0.5 mg/kg IV dose), ketamine has been shown to have better analgesic property than opiates for the acute and chronic pain*.

What was the research question?

*What is the efficacy of low-dose ketamine for the control of acute pain in emergency setting?*

What was the major finding of the study?

*The pooled standard mean difference of ketamine for pain score was* −*0.45 (95%CI:* −*0.84 to* −*0.07; p<0.001) within 30 minutes*.

How does this improve population health?

*The study provides important information to clinicians and emergency physicians on the use of low-dose ketamine for management of acute pain in emergency setting*.

#### Outcome Measures

Our outcome measures were pain score, the need for rescue analgesic medication, and adverse effects (gastrointestinal, neurological, psychological or cardiopulmonary side effects). Studies reporting either the pain score or need for rescue analgesic medication were included.

### Search Strategy

We systematically searched electronic databases, including PubMed Central, EMBASE, MEDLINE, and the Cochrane Library and search engines such as ScienceDirect and Google Scholar, for eligible studies using medical subject headings and free-text words. Individual search results were combined, and the final search was performed using appropriate Boolean operators (“OR” and “AND”) and narrowed down using the available filters on time period (from inception to October 2021), language (English language only), as summarized in the Supplementary Appendix.

### Study Selection

We selected the relevant studies by screening the title, abstract, and keywords of the identified manuscripts. For the studies that met the eligibility criteria, we then reviewed and screened the full-text articles were. The eligibility criteria of the reviews were assessed. We included studies that met eligibility criteria with respect to design, participants, intervention, comparisons, and outcomes. All cases of disagreement were resolved by discussion.

### Data Extraction

Data was manually extracted using a predefined structured data extraction form and included authors, title of study, year of publication, study period, study design, setting, country/region, total sample size, outcome assessment details, average age, and primary and secondary outcomes in each approach. The primary investigator was responsible for entering the data, and the secondary investigator double-checked for accuracy.

### Risk-of-bias (Quality) Assessment

Quality of included studies was assessed by two independent investigators using the revised Cochrane risk-of-bias tool (RoB 2) for RCTs.^15^ We assessed risk of bias under the following domains:

- **Domain 1:** Bias risk arising from the process of randomization
- **Domain 2:** Bias risk due to deviation from the intended intervention
- **Domain 3:** Bias risk arising due to missing data on outcomes
- **Domain 4:** Bias risk in the measurement of outcome
- **Domain 5:** Bias risk in the selection of reported result.

Based on the rating obtained from these domains, we classified the quality of evidence of each study as having “low bias risk,” “high bias risk,” and “some concerns.”

### Statistical Analysis

We performed data analysis using STATA version 14.2. (StataCorp LLC, College Station, TX). For continuous data such as pain score and total analgesic requirement, we obtained mean, standard deviation, and total sample size for both groups. The pooled effect was calculated as standardized mean difference (SMD) with 95% confidence interval, as different scales were used by each of the studies for reporting pain scores. Since all the other outcomes were dichotomous, the number of events and participants in each group were entered to obtain the pooled effect estimate as a risk ratio (RR) with 95% CI. Visual representation of these pooled estimates was done by forest plot. We used the random-effects model with inverse variance method to calculate the weight of individual studies.^16^

Heterogeneity was evaluated by chi square of heterogeneity and the I^2^ statistic. A P-value less than 0.05 in chi square testing indicated significant heterogeneity, while we used the I^2^ value to quantify the heterogeneity using the following criteria: less than 25% = mild heterogeneity, 25–75% = moderate heterogeneity and >75% = substantial heterogeneity.^16^ We performed subgroup analysis and meta-regression to explore the source of heterogeneity using possible potential covariates such as dose of ketamine and comparison group. Publication bias was evaluated and visually represented using a funnel plot. We assessed the asymmetry of plot using Egger’s test. A *P*-value < 0.10 was considered as statistically significant publication bias.^17^

### Quality of Evidence

The risk of bias and quality of evidence for included studies were independently assessed by two investigators using Grading of Recommendations Assessment, Development and Evaluation (GRADE) guidelines.^16^ The GRADE approach consists of five components: 1) risk of bias assessment; 2) indirectness; 3) imprecision; 4) inconsistency; and 5) publication bias.

**Risk of bias assessment:** Determined using the Cochrane risk-of-bias tool

**Indirectness:** Assessed in terms of population, intervention, comparison, or outcomes

**Imprecision:** Determined the precision of the estimate obtained, based on sample size and CI

**Inconsistency**: Assessed evidence of heterogeneity using the I^2^ statistic and chi square test of heterogeneity

**Publication bias:** Assessed using Egger’s test and a funnel plot.

Finally, we classified the quality of the included studies as “very low,” “low,” “moderate,” and “high” based on certainty of evidence.

## RESULTS

### Study Selection

**Figure 1:**
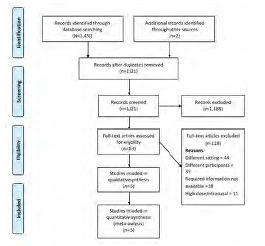
PRISMA flowchart. *PRISMA*, Preferred Reporting Items for Systematic Reviews and Meta-Analyses.

Figure 1 shows the PRISMA flowchart of the study selection process. During primary screening, 188 full-text studies were retrieved. Of them, 133 studies remained after removal of duplicates. An additional three articles were retrieved from the bibliography of the screened articles. Studies underwent secondary screening that resulted in a total of 15 studies with 1,613 participants, which satisfied the inclusion criteria and were included in the analysis.^9^–^11^,^18^–^29^

### Study Characteristics

We have included only RCTs in our review. Most studies (8/15) were conducted in the United States of America (US), followed by Middle Eastern countries such as Iran and Saudi Arabia. The mean age of study participants in the intervention arm ranged from 29.1–77.3 years, while in the control arm it ranged from 29.6–77.1. The sample sizes among the included studies varied from 34–300. The IV dose of ketamine ranged from 0.1–0.5 mg/kg. Morphine was the most commonly used opioid in the comparison group (10 studies) followed by placebo (normal saline in five studies). Regarding quality assessment, at least half of the studies had higher risk of bias (seven studies), while the remaining studies evidenced some concerns as per the RoB 2 checklist (Table 1).

### Efficacy of Ketamine for Control of Acute Pain

#### Pain Score within 15 Minutes

**Figure 2:**
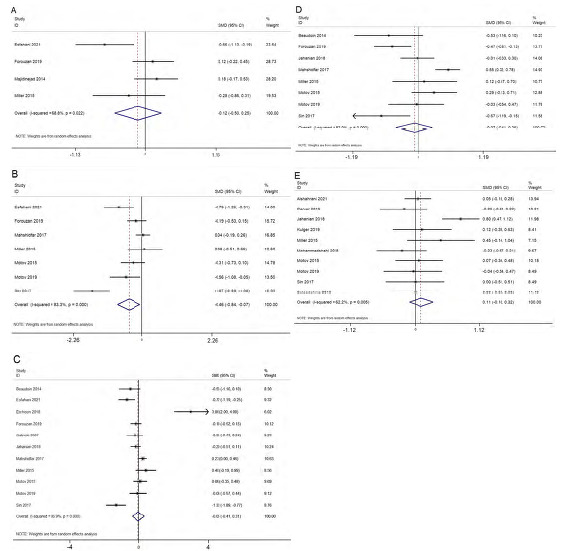
Forest plot showing the difference in pain score between ketamine and control group A) within 15 minutes, B) within 30 minutes, C) within 45 minutes, D) within 60 minutes, and E) > 60 minutes.

Four studies reported on the difference in pain score within 15 minutes. The pooled SMD was −0.12 (95% CI −0.50–0.25; I^2^=68.8%), indicating no significant difference between the ketamine and control groups in the control of pain within 15 minutes (Figure 2A). Analysis based on the dose of ketamine was not possible as each of the studies used different doses, making it difficult to provide a pooled estimate for each dose. Similarly, analysis based on the control group was not possible as all the studies used morphine in the control group. The quality of evidence was found to be low as per the GRADE approach.

#### Pain Score within 30 Minutes (15–29 minutes)

Seven studies reported on the difference in pain score within 30 minutes. The pooled SMD was −0.45 (95% CI −0.84–−0.07; I^2^=83.3%), indicating a significant decline in the pain score among the patients’ receiving ketamine when compared to the control arm within 30 minutes (Figure 2B). Sensitivity analysis did not affect the significant findings obtained in the primary analysis in terms of magnitude or direction of association because of small-study effects (Supplementary Figure 1). The most commonly used dose of ketamine (five studies) was 0.3 mg/kg and was associated with a significant difference in pain score (pooled SMD = −0.51, 95% CI −1.01–−0.01). Analysis based on the control group was not possible as all the studies (except Sin et al 2017) used morphine as control group. The quality of evidence was found to be low as per the GRADE approach.

#### Pain Score within 45 Minutes (30–44 minutes)

Eleven studies reported on the difference in pain score within 45 minutes. The pooled SMD was −0.05 (95% CI −0.41–0.31; I^2^=86.9%), indicating no significant difference in pain score between ketamine and the control group within 45 minutes (Figure 2C). Sensitivity analysis did not reveal any significant difference in the magnitude or direction of association because of small-study effects (Supplementary Figure 2). The funnel plot showed a symmetrical plot indicating the lack of publication bias (Supplementary Figure 3), and it was further confirmed by a non-significant Egger’s test (*P*=0.96). The quality of evidence was found to be low as per the GRADE approach.

Analysis based on the dose of ketamine did not show significant effect at any of the doses ranging from 0.1–0.5 mg/kg. Analysis based on the control group did not reveal a significant effect for ketamine when compared to morphine (pooled SMD = −0.11; 95% CI −0.3–0.13) or placebo (pooled SMD = 0.42, 95% CI −1.51–2.36). Univariable meta-regression revealed that none of these factors were responsible for the significant heterogeneity in the estimates. The quality of evidence was found to be low as per the GRADE approach.

#### Pain Score between 45–60 Minutes

Eight studies reported on the difference in pain score between 45–60 minutes. The pooled SMD was −0.07 (95% CI −0.41 to 0.26; I^2^=82%), indicating no significant difference in pain score between the ketamine and control groups between 45–60 minutes (Figure 2D). Sensitivity analysis did not reveal any significant difference in the magnitude or direction of association because of small-study effects (Supplementary Figure 4). Analysis based on the dose of ketamine did not show significant effect at any of the doses ranging from 0.1–0.5 mg/kg. Analysis based on the control group was not possible as all the studies (except Sin et al 2017) used morphine in the control group. The quality of evidence was found to be low as per the GRADE approach.

#### Pain Score after 60 Minutes

Eight studies reported on the difference in pain score after 60 minutes. The pooled SMD was 0.17 (95% CI −0.07–0.42; I^2^=64.8%), indicating no significant difference in pain score between the ketamine group and control group after 60 minutes (Figure 2E). Sensitivity analysis did not reveal any significant difference in the magnitude or direction of association because of small-study effects (Supplementary Figure 5). Analysis based on the dose of ketamine was not possible as each of the studies used a different dose, making it difficult to provide a pooled estimate for each dose. Analysis based on the control group did not reveal a significant effect for ketamine when compared to morphine (pooled SMD = 0.28, 95% CI 0.05–0.62) or placebo (pooled SMD = −0.05, 95% CI −0.32–0.22). The quality of evidence was found to be low as per the GRADE approach.

#### Need for Rescue Analgesic Medication

**Figure 3:**
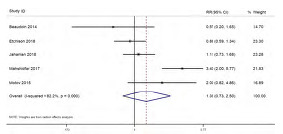
Forest plot showing the difference in need for rescue analgesic between the ketamine and control groups.

Five studies reported on the difference in need for rescue analgesic medication between the ketamine and control groups. The pooled RR was 1.35 (95% CI 0.73–2.50; I^2^=82.2%), indicating no significant difference in need for rescue analgesics between the ketamine and control groups (Figure 3). Sensitivity analysis did not reveal any significant difference in the magnitude or direction of association because of small-study effects (Supplementary Figure 6). Analysis based on the dose of ketamine did not show significant effect at any of the doses ranging from 0.1–0.5 mg/kg. Analysis based on the control group was not possible as all the studies (except Etchison et al 2018) used morphine in the control group. The quality of evidence was found to be low as per the GRADE approach.

### Adverse Effects

#### Gastrointestinal Side Effects

**Figure 4:**
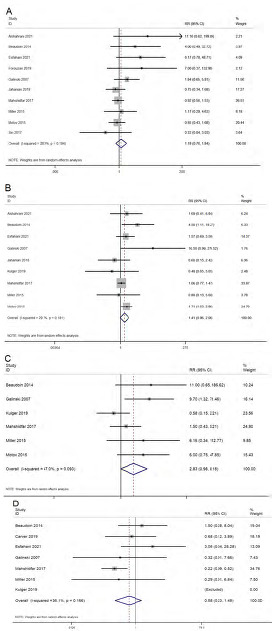
Forest plot showing the difference in adverse reactions between the ketamine and control groups. A) gastrointestinal side effects, B) neurological side effects, C) psychological side effects, and D) cardiopulmonary side effects.

Ten studies reported on the difference in gastrointestinal side effects (nausea and vomiting) between the ketamine and control groups. The pooled RR was 1.18 (95% CI 0.76–1.84; I^2^=28.3%), indicating no significant difference in the gastrointestinal side effects between the ketamine and control groups (Figure 4A). Analysis based on the dose of ketamine did not show any difference in gastrointestinal side effects at any of the doses ranging from 0.1–0.5 mg/kg. Analysis based on the control group revealed no significant difference compared to any of the control group. The quality of evidence was found to be low as per the GRADE approach.

#### Neurological Side Effects

Ten studies reported on the difference in neurological side effects (dizziness, drowsiness, emergence phenomena, dysphoria/dissociation) between the ketamine and control groups. The pooled RR was 1.41 (95% CI 0.96–2.06; I^2^=29.7%), indicating no significant difference in the neurological side effects between the ketamine and control groups (Figure 4B). Analysis based on the dose of ketamine showed significantly higher risk of neurological side effects at a dose of 0.3 mg/kg (pooled RR=1.82, 95% CI 1.17–2.83), while it was not significant at a dose less than 0.3 mg/kg. Analysis based on the control group revealed no significant difference compared to any of the control group. The quality of evidence was found to be low as per the GRADE approach.

#### Psychological Side Effects

Six studies reported on the difference in psychological side effects (delirium, hallucinations, and mood changes) between the ketamine and control groups. The pooled RR was 2.83 (95% CI 0.98–8.18; I^2^=47%), indicating no significant difference in psychological side effects between the ketamine and control groups (Figure 4C). Subgroup analysis based on the route of administration, dose, or control group did not reveal any difference in terms of magnitude or direction of association. The quality of evidence was found to be low as per the GRADE approach.

#### Cardiopulmonary Side Effects

Seven studies reported on the difference in cardiopulmonary side effects (hypoxia, hypotension, and respiratory failure) between the ketamine and control groups. The pooled RR was 0.58 (95% CI 0.23–1.48; I^2^=36.1%), indicating no significant difference in cardiopulmonary side effects between the ketamine and control groups (Figure 4D). Subgroup analysis based on the route of administration, dose, or control group did not reveal any difference in terms of magnitude or direction of association. The quality of evidence was found to be low as per the GRADE approach.

## DISCUSSION

Our aim in this systematic review was to obtain a comprehensive estimate of the efficacy and safety of low-dose ketamine for the control of acute pain among patients presenting to the ED. We found that ketamine causes a significant decline in the pain score within 30 minutes of infusion when compared to any control group. We also found that ketamine had maximum efficacy at a dosage of 0.3 mg/kg when administered through the IV route. However, primary evidence is limited on the dose-related analysis and conclusive evidence on dosage cannot be provided. Ketamine had better efficacy when compared to morphine and placebo. However, its effect was similar to morphine and placebo at other time intervals (>30 minutes). Previous reviews have also reported that ketamine had equivalent or higher efficacy in the pain score at short-term and long-term time intervals compared to opioids such as morphine or fentanyl.^12^,^13^,^30^ In addition, a review has also reported 0.3 mg/kg as the optimal dose of ketamine.^13^

Mechanism of action of ketamine involves binding to the spinal μ receptors and increasing the efficacy of the opioid-induced signalling.^31^ In addition, ketamine also functions as a NMDA receptor antagonist and acts preferentially post-synoptically, causing a reduction in hyperexcitability.^32^ Therefore, the blockage of NMDA by ketamine might further improve the opioid efficacy leading to opioid-sparing effect.

We also assessed risk of various adverse effects associated with ketamine for the management of acute pain in emergency settings. We found that ketamine was associated with a significantly higher risk of neurological side effects at a dose of 0.3 mg/kg when compared to the opioid group of drugs. Previous reviews have also warned against the neurological complications of ketamine, especially at higher doses. Ketamine prevents serious adverse effects of opioids and inhibits the chronic pain that develops due to opioid tolerance.^33^,^34^ Ketamine inhibits nociception through the high affinity and selective interaction with the NMDA receptor.^33^,^34^ At the full-anaesthetic dose, ketamine activates different types of opioid receptors, such as κ, μ, and σ opioid receptors, with various affinities.^35^,^36^ However, previous review has shown that a higher rate of neurological effects was associated with the intranasal route.^37^,^38^ Other adverse effects such as gastrointestinal, psychological, and cardiopulmonary effects were almost equivalent between the ketamine and opioid groups.

The major strength of this paper is that it provides an up-to-date, comprehensive review of the efficacy and safety of low-dose ketamine in the management of acute pain among patients presenting to the emergency setting. This review also includes a large number of studies to provide a reasonable estimate on burden. We performed additional sensitivity analysis, subgroup analysis, and meta-regression to provide a more robust estimate. We did not find significant publication bias, adding more credibility to the results.

## LIMITATIONS

Our review did have certain limitations. Almost half of the included studies had high risk of bias. The chi square test for heterogeneity also revealed significant variability across the included studies. This could have led to biased estimates with limited generalizability. We tried to overcome this limitation by performing meta-regression. However, meta-regression could not be performed for the majority of the outcomes due to the limitation in the number of studies (<10 studies). Ketamine works fairly quickly, yet only four studies reported pain scores within 15 minutes, and only seven studies reported them within 30 minutes. This was a limitation with the primary studies, in that it was not possible to really report the full pain effect of ketamine. Finally, we could not perform subgroup analysis based on the type of pain. Future studies could specifically focus on different types of pain conditions, as the mechanism and neurochemistry behind the pain pathways for each condition is entirely different.

## CONCLUSION

Low-dose ketamine may have equivalent or higher efficacy and safety when compared to opioids for managing acute pain among patients presenting to the emergency care setting. However, we could not make a conclusive recommendation based on the available evidence. Hence, further studies are required to compare the combination of ketamine with specific opioids to identify the best approach to pain control. This will help clinicians manage their patients with the least chance of complications and an optimal success rate.

## Supplementary Information















## Figures and Tables

**Table 1:** Characteristics of the included studies (N=28).

Author and year	Country	Sample size	Study participants	Pain scale	Route of administration of ketamine	Dose of ketamine	Comparator group	Mean age	Risk of bias
Alshahrani 2021	Saudi Arabia	I=138 C=140	Adults with sickle cell disease who presented with acute sickle vaso-occlusive crisis.	NRS	Intravenous	0.3 mg/kg	Morphine	I=29.1 C=29.6	High
Beaudoin 2014	USA	I=20 C=20	Patients 18–65 years old with acute moderate to severe pain and pain duration <7 days) who were deemed by their treating physician to require IV opioid	NRS	Intravenous	0.3 mg/kg	Morphine	I=37.5 C=32.5	Some concerns
Carver 2019	USA	I=45 C=46	Adult patients with three or more rib fractures admitted to a Level I trauma center	NRS	Intravenous	2.5 μg/kg	Placebo	I=46 C=50	High
Esfahani 2021	Iran	I=36 C=37	Patients referred to EDs due to isolated limb traumatic injuries	NRS	Intravenous	0.1 mg/kg	Morphine	I=32.5 C=33.4	High
Etchison 2018	USA	I=16 C=18	Adults 18–65 years of age with acute migraine at a single academic ED	NRS	Intravenous	0.2 mg/kg	Placebo	I=38.5 C=30.5	Some concerns
Forouzan 2019	Iran	I=68 C=68	Patients who had suffered kidney pain due to kidney stones referred to Ahvaz Imam Khomeini Hospital	NRS	Intravenous	0.3 mg/kg	Morphine	NR	High
Galinski 2007	France	I=33 C=32	Trauma patients with a severe acute pain defined as a VAS score of at least 60/100 were enrolled	VAS	Intravenous	0.2 mg/kg	Placebo	I=35 C=40	Some concerns
Jahanian 2018	Iran	I=78 C=78	Adult patients 18–65 years with upper or lower extremity long bone fractures caused by blunt trauma referring to our ED	VAS	Intravenous	0.5 mg/kg	Morphine	I=35.8 C=36.3	High
Kugler 2019	USA	I=30 C=29	Elderly patients (age, ≥65 years) with three or more rib fractures admitted to a Level I trauma center	NRS	Intravenous	0.2 mg/kg	Placebo	I=75 C=73	High
Mahshidfar 2017	Iran	I=150 C=150	Trauma patients aged 18–70 years with a musculoskeletal pain score of 5 or more on 11-point NRS who were referred to EDs	NRS	Intravenous	0.2 mg/kg	Morphine	I=34.4 C=34.1	Some concerns
Majidinejad 2014	Iran	I=63 C=63	Patients with fractures of long bones, referred to the emergency unit.	NRS	Intravenous	0.5 mg/kg	Morphine	I=35.1 C=53.6	Some concerns
Miller 2015	USA	I=24 C=21	Patients 18–59 years with acute abdominal, flank, low back, or extremity pain were enrolled	NRS	Intravenous	0.3 mg/kg	Morphine	I=31 C=29	Some concerns
Motov 2015	USA	I=45 C=45	ED patients 18–55 years and experiencing moderate to severe acute abdominal, flank, or musculoskeletal pain	NRS	Intravenous	0.3 mg/kg	Morphine	I=35 C=36	Some concerns
Motov 2019	USA	I=30 C=30	ED patients 18–55 years and experiencing moderate to severe acute abdominal, flank, or musculoskeletal pain	NRS	Intravenous	0.3 mg/kg	Morphine	I=77.3 C=77.1	Some concerns
Sin 2017	USA	I=30 C=30	Patients >18 years who presented to the ED with a chief complaint of acute pain with moderate to severe intensity	NRS	Intravenous	0.3 mg/kg	Placebo	I=41 C=48	High

*I*, Intervention (ketamine) group; *C*, control/comparator group; *FPS*, Faces Pain Scale; *NR*, not reported; *NRS*, Numerical Rating Scale; *VAS*, Visual Analog Scale; *USA*, United States of America; *ED*, emergency department.
