# Supplementary material for: Efficacy of Low-dose Ketamine for Control of Acute Pain in the Emergency Setting: A Systematic Review and Meta-analysis of Randomized Controlled Trials
Source: West J Emerg Med. 2023 May 9;24(3):644–53. doi: 10.5811/westjem.2023.2.58368 (PMC10284511; doi:10.5811/westjem.2023.2.58368)
Supplement: Supplementary file 1 [file wjem-24-644-SupplementaryAppendix.pdf]

SEARCH STRATEGY:

EMBASE:

('emergency care'/exp OR 'acute care' OR 'acute medical care' OR 'emergency care' OR 'emergency health care' OR 'emergency medical care' OR 'patient care, prehospital' OR 'pre-hospital care' OR 'pre-hospital patient care' OR 'prehospital care' OR 'prehospital patient care') AND ('ketamine'/exp OR '2 (2 chlorophenyl) 2 (methylamino) cyclohexanone' OR '2 (2 chlorophenyl) 2 (methylamino) cyclohexanone hydrochloride' OR '2 (2 chlorophenyl) 2 methylaminocyclohexanone' OR '2 (methylamino) 2 (2 chlorophenyl) cyclohexanone' OR '2 (ortho chlorophenyl) 2 (methylamino) cyclohexanone' OR '2 (ortho chlorophenyl) 2 methylaminocyclohexanone' OR '2 (ortho chlorophenyl) 2 methylaminocyclohexanone hydrochloride' OR '2 methylamino 2 (2 chlorophenyl) cyclohexanone' OR '2 ortho chlorophenyl 2 methylaminocyclohexanone' OR 'anesject' OR 'calipsol' OR 'calypsol' OR 'ci 581' OR 'ci581' OR 'cl 369' OR 'cl369' OR 'cn 52, 372 2' OR 'cn 52372 2' OR 'cn 523722' OR 'cn52, 372 2' OR 'cn52372 2' OR 'cn523722' OR 'imalgene' OR 'kalipsol' OR 'katamine' OR 'keta-hameln' OR 'ketaject' OR 'ketalar' OR 'ketalin' OR 'ketamax' OR 'ketamine' OR 'ketamine hcl' OR 'ketamine hydrochloride' OR 'ketaminol vet' OR 'ketanest' OR 'ketased' OR 'ketaset' OR 'ketaved' OR 'ketavet' OR 'ketmin' OR 'ketoject' OR 'ketolar' OR 'narkamon' OR 'narketan' OR 'soon-soon' OR 'tekam' OR 'velonarcon' OR 'vetalar') AND ('pain'/exp OR 'acute pain' OR 'deep pain' OR 'lightning pain' OR 'nocturnal pain' OR 'pain' OR 'pain response' OR 'pain syndrome' OR 'treatment related pain') AND ('randomized controlled trial'/exp OR 'controlled trial, randomized' OR 'randomised controlled study' OR 'randomised controlled trial' OR 'randomized controlled study' OR 'randomized controlled trial' OR 'trial, randomized controlled') – **51 results**

PubMed Search:

(((("Ketamine"[MeSH Terms] AND "Acute Pain"[MeSH Terms]) OR "Pain Management"[MeSH Terms] OR "Pain"[MeSH Terms]) AND "Randomized Controlled Trial"[Publication Type]) OR "Randomized Controlled Trials as Topic"[MeSH Terms]) AND "emergency service, hospital"[MeSH Terms] – **921 results**
