# Supplementary figures and images for: Efficacy of Low-dose Ketamine for Control of Acute Pain in the Emergency Setting: A Systematic Review and Meta-analysis of Randomized Controlled Trials
Source: West J Emerg Med. 2023 May 9;24(3):644–53. doi: 10.5811/westjem.2023.2.58368 (PMC10284511; doi:10.5811/westjem.2023.2.58368)

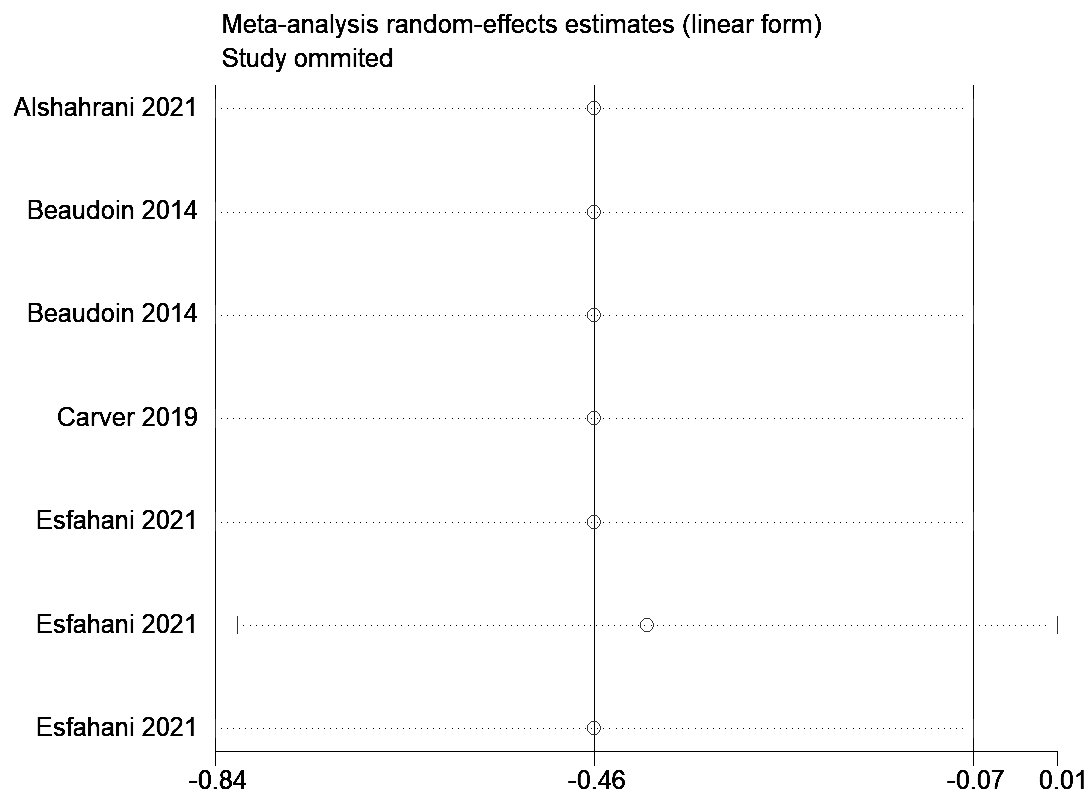

Supplement: Supplementary file 2 [file wjem-24-644_Supplementary_Figure_1.tif]

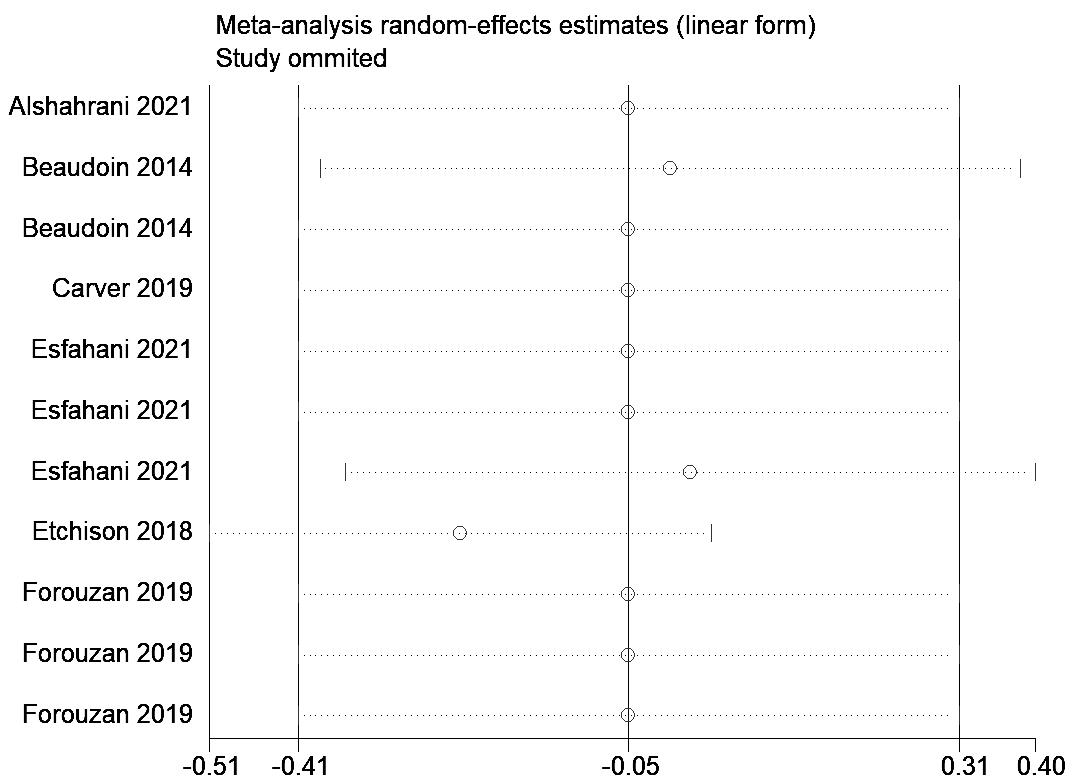

Supplement: Supplementary file 3 [file wjem-24-644_Supplementary_Figure_2.tif]

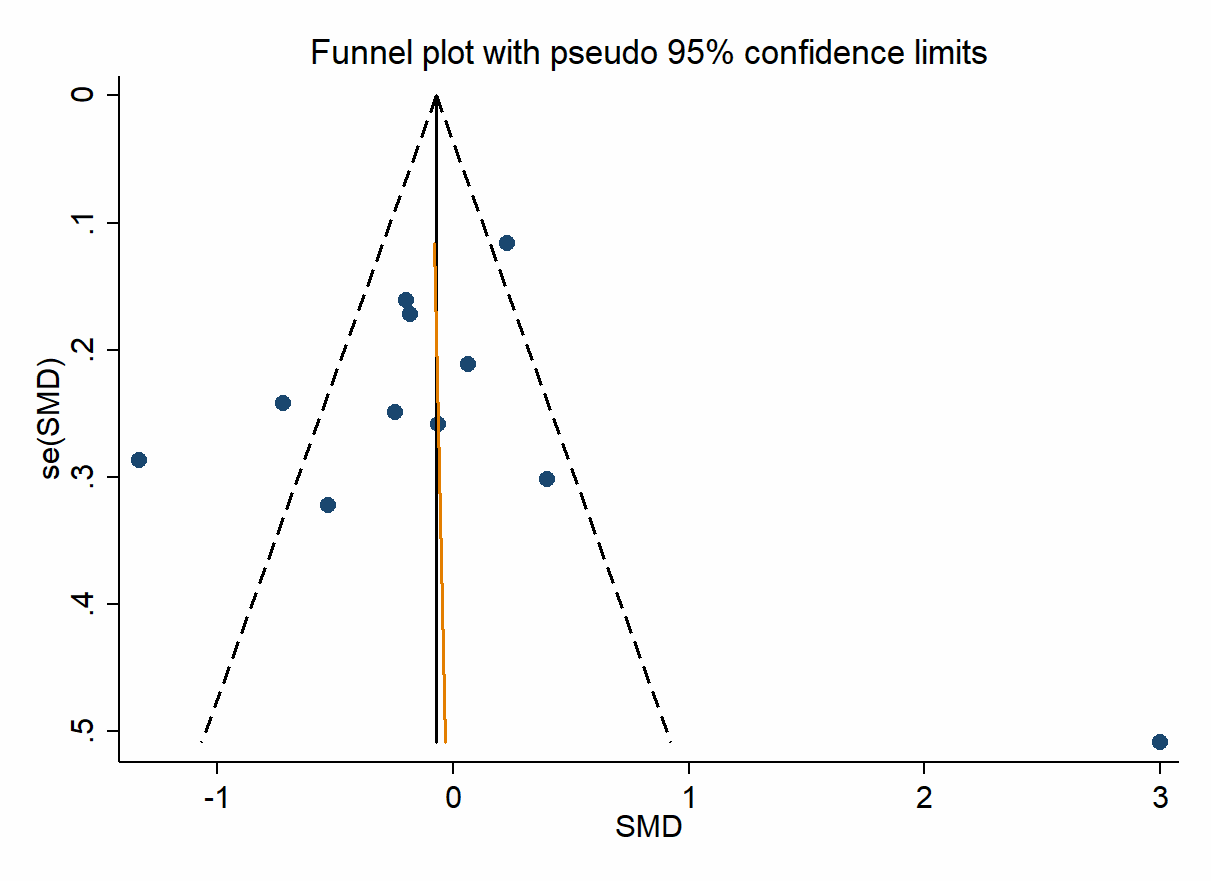

Supplement: Supplementary file 4 [file wjem-24-644_Supplementary_Figure_3.tif]

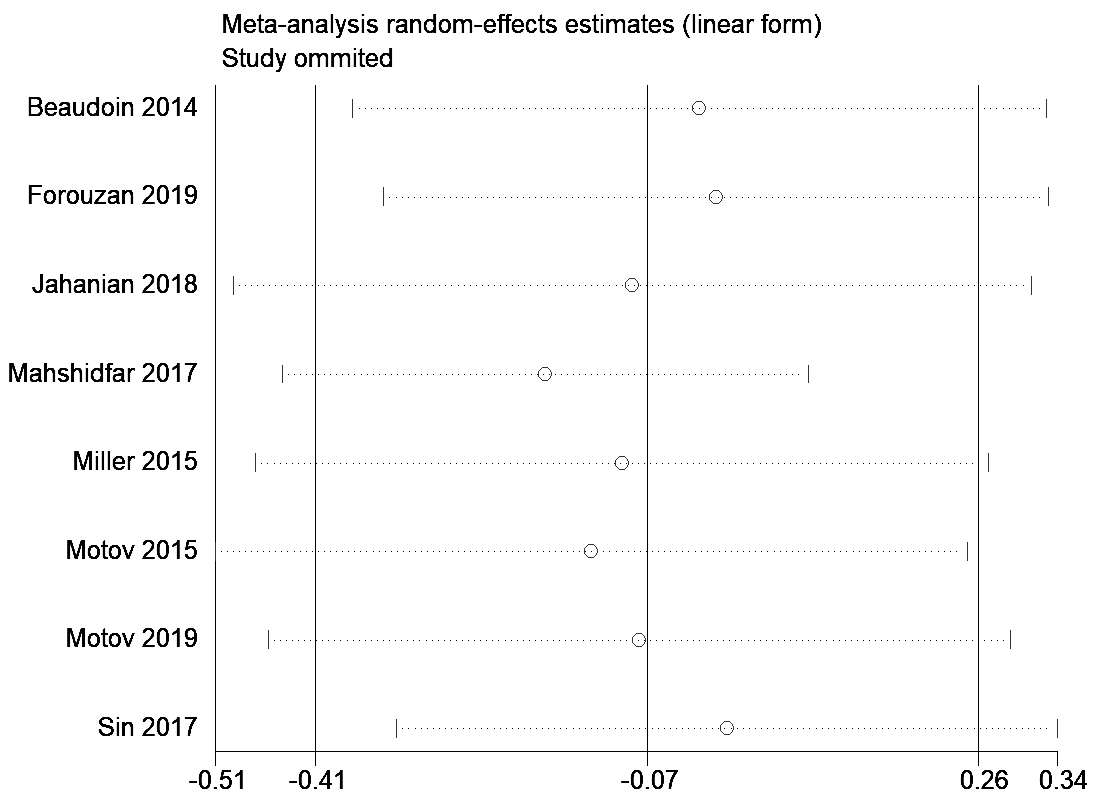

Supplement: Supplementary file 5 [file wjem-24-644_Supplementary_Figure_4.tif]

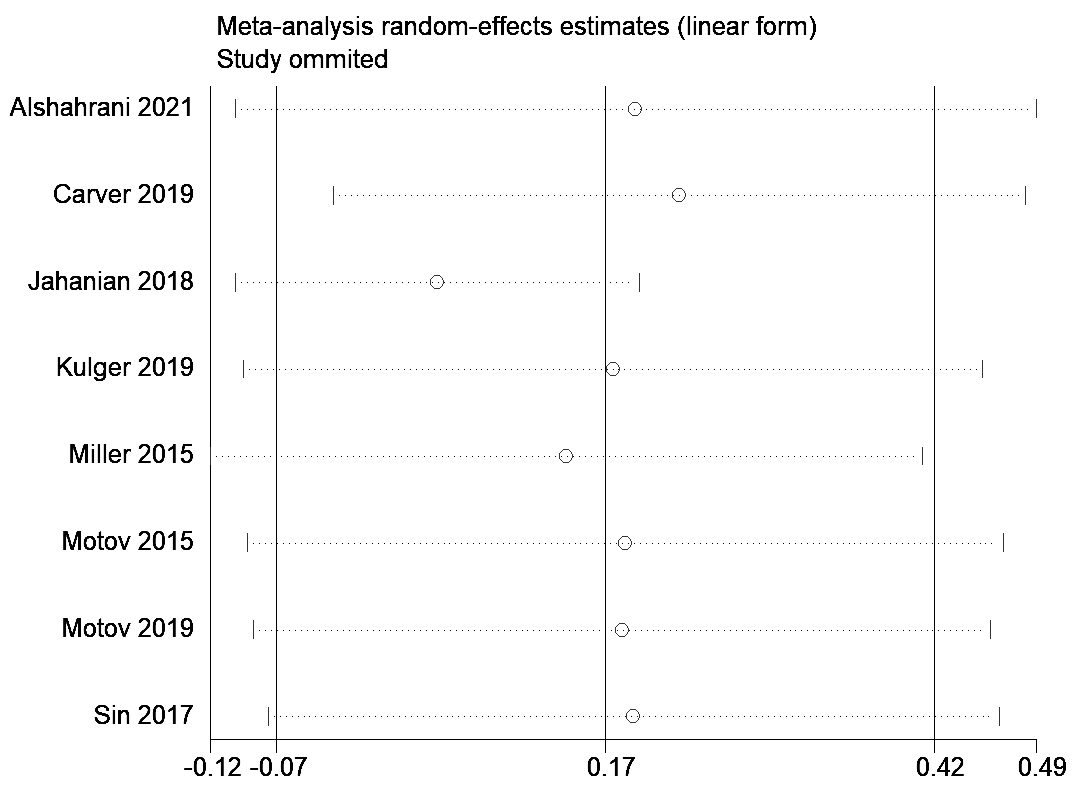

Supplement: Supplementary file 6 [file wjem-24-644_Supplementary_Figure_5.tif]

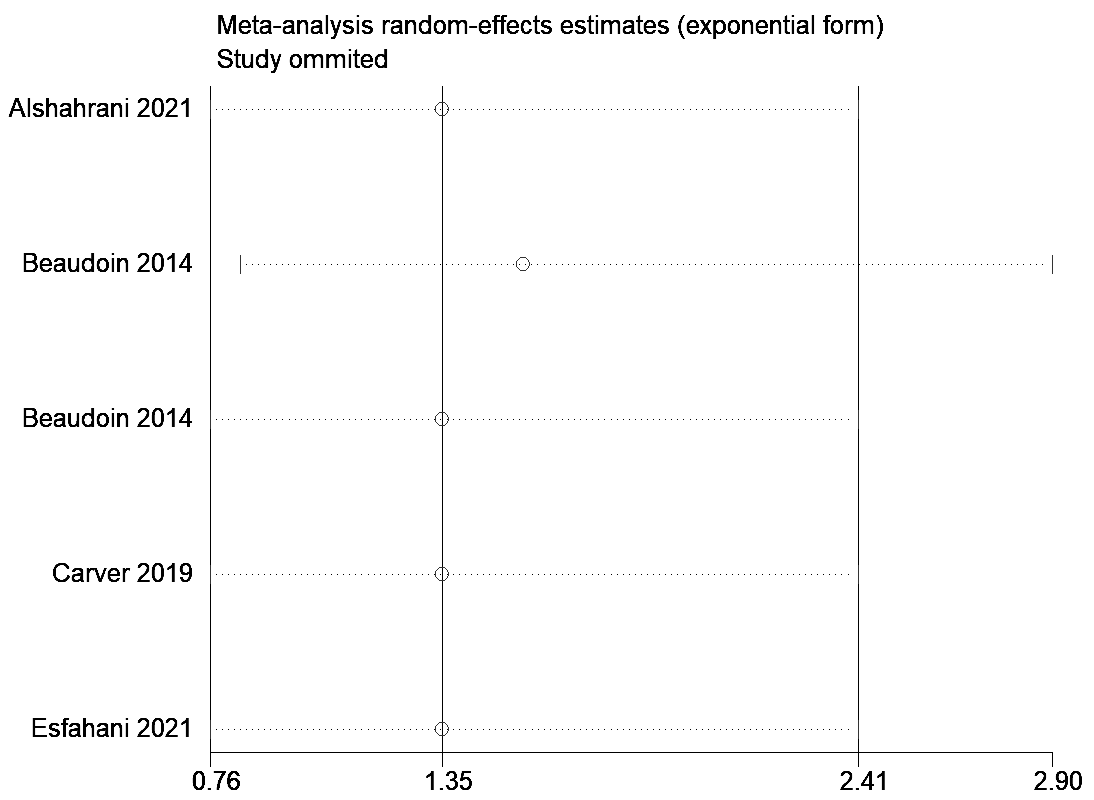

Supplement: Supplementary file 7 [file wjem-24-644_Supplementary_Figure_6.tif]
